# The Influence of Emotional Intelligence on Job Burnout of Healthcare Workers and Mediating Role of Workplace Violence: A Cross Sectional Study

**DOI:** 10.3389/fpubh.2022.892421

**Published:** 2022-05-11

**Authors:** Yiyin Cao, Lei Gao, Lihua Fan, Mingli Jiao, Ye Li, Yuanshuo Ma

**Affiliations:** School of Health Management, Harbin Medical University, Harbin, China

**Keywords:** emotional intelligence, job burnout, workplace violence, healthcare workers, measure

## Abstract

**Objective:**

Globally, reducing job burnout among healthcare workers is considered a basic healthcare policy goal. Emotional intelligence, as an essential protective factor against psychosocial risks and a measurable positive psychological resource, still receives less attention in the process of reducing job burnout among healthcare workers. This study aims to explore the level of job burnout among healthcare workers who are victims of workplace violence in China, to examine the influence of emotional intelligence on job burnout among healthcare workers, and to verify the mediating role of workplace violence; furthermore, providing a new perspective for health organizations and hospital administrators in order to relieve the level of job burnout and workplace violence among healthcare workers.

**Methods:**

A cross-sectional study was used to collect data from six tertiary public hospitals in three provinces (cities) in Eastern (Shandong and Tianjin) and Western (Gansu) China in 2018, which are large healthcare sites providing care to patients upon referral from primary and secondary hospitals. A total of 2,450 questionnaires were distributed, with 2,061 valid questionnaires and a valid return rate of 88.95%. Of these, 825 healthcare workers had experienced workplace violence, accounting for 40.03% of the sample. A descriptive analysis, univariate analysis, Pearson correlation analysis, and mediated regression analysis were used to assess the level of job burnout among healthcare workers who are victims of violence, the effect of emotional intelligence on job burnout, and the mediating role of workplace violence.

**Results:**

The mean job burnout score of the healthcare personnel who were victims of violence was 35.56, with 70% suffering from moderate and high burnout. The emotional intelligence of healthcare workers is significantly negatively correlated with the degree of job burnout (Emotional exhaustion: *r* = 0.18, *p* < 0.01, Depersonalization: *r* = 0.24, *p* < 0.01, Reduced personal achievement: *r* = 0.24, *p* < 0.01) and workplace violence frequency (*r* = −0.22, *p* < 0.01). Further, workplace violence has a partial mediating effect on emotional intelligence and the two dimensions of job burnout (emotional exhaustion and depersonalization).

**Conclusions:**

This study is the first to combine emotional intelligence level, experiences of workplace violence, and job burnout levels of healthcare workers. We suggest that improving the emotional intelligence of healthcare staff has practical significance in reducing the level of job burnout directly and will reduce the incidence of burnout by reducing the frequency of violence (especially for emotional exhaustion and depersonalization). We provide specific and effective strategies for developing and guiding healthcare workers in the healthcare sector based on emotional intelligence.

## Introduction

Job burnout is defined as a state of physical and emotional exhaustion caused by excessive and sustained levels of work-related stress (1). In general, job burnout has three key dimensions: feelings of over-extension and the depletion of resources (emotional exhaustion), negative or callous responses to job responsibilities (depersonalization), and feelings of incompetence and a lack of achievement (decreased personal accomplishment)(2). Researchers and administrators are increasingly recognizing the problem of job burnout as a major international concern in the field of occupational health (3, 4). In the medical field, the detrimental effects of job burnout are important not only for the psychological well-being of physicians but also for healthcare organizations because they increase absenteeism (5), job dissatisfaction (6), and willingness to leave (7). Reducing job burnout has been recognized as a fundamental healthcare policy goal across the globe. Scholars have found through bibliometrics that developing countries may neglect occupational stress in order to devote more energy to physical health issues, and that job burnout is one of the top three occupational stressors (8). Thus, continuous attention to the incidence of job burnout among health workers in China is of great significance in improving the quality of medical services and related reforms.

Most studies in the literature related to job burnout in healthcare professionals have used a disease-based mode (9) to analyze the harm or consequences of burnout. The prevailing negative bias is illustrated by the fact that the total number of publications on negative states exceeds that of positive states by a ratio of 14: 1 (10). However, psychologists have gradually discovered that by focusing on pain, people may experience more pain. At the same time, modern psychology also encourages scholars to conduct research from a more positive perspective (11). Particularly for healthcare workers who have been exposed to interpersonal pressure for a long time and have experienced dynamic changes between job burnout and mental health, it is particularly important to use positive psychology to study how to reduce their job burnout level. Meanwhile, the importance of non-technical skills is increasingly emphasized in current research on doctor-patient relationship (12). Therefore, emotional intelligence (EI), as a measurable positive psychological resource and a key non-technical skill, should receive attention in the process of mitigating burnout levels in healthcare workers. Mayer and Salovey define emotional intelligence as the ability to recognize, understand, and regulate one's own and others' emotions, to differentiate between them, and to use this information to guide thoughts and actions (13). This concept of emotional intelligence contains the following four dimensions: (1) Self-emotion Appraisal, (2) Others Emotion Appraisal, (3) Regulation of Emotion, and (4) Uses of Emotion. Further, the concept aims to capture individual differences in the way people deal with emotions. As such, emotional intelligence is a prerequisite for key skills such as communication, self-confidence, empathy, creativity, sensitivity, self-awareness, and self-control (14). Studies have found that emotional intelligence is related to an individual's subjective well-being (15), way of dealing with problems (16), and intuition about stress (17). As research on emotional intelligence continues, it is also gradually being incorporated into the field of medical research in the form of empirical studies and has been shown to correlate with patient satisfaction, the occurrence of medical errors, communication skills, and the performance of medical staff (18, 19). Although emotional intelligence plays an important and profoundly promising role, it is surprising that few studies have examined the relationship between emotional intelligence and job burnout among healthcare workers in medical practice.

In addition, according to emotional intelligence theory, individuals with high emotional intelligence are able to identify their own emotions and the emotions of others, express emotions in a socially acceptable way, understand the causes and consequences of emotions, use them to enhance their thoughts, actions, and social relationships, and regulate them when they are inappropriate for their goals or situational contexts. Goleman (20) proposed that, by possessing EI, a person can manage painful emotions and control impulses in situations involving conflict, which in turn affects the management of interference behaviors and violent incidents. Thus, it is reasonable to speculate on the correlation between the frequency of exposure to violence in healthcare populations and the emotional intelligence of individuals. The World Health Organization (WHO) defines workplace violence as physical or psychological “incidents where staff are abused, threatened, or assaulted in circumstances related to their work, including commuting to and from work, and involves an explicit or implicit challenge to their safety, well-being, or health (21). Healthcare workers are 16 times more likely to experience workplace violence than workers in other occupations (22). Itzhaki et al. (23) found that almost 90% of the healthcare worker population is exposed to violence. At the same time, scholars generally agree that WPV is still increasing in the health service sector (24) and some studies have confirmed that severe medical violence can even lead to post-traumatic stress disorder (PTSD) and induce severe job burnout in clinical health care workers (25). When the job burnout of healthcare workers injured by violent incidents increases, the quality of medical services is affected, and the relationship between doctors and patients becomes more tense, forming a vicious circle. This global, social phenomenon is no longer a simple doctor-patient relationship problem, but a serious social problem that needs urgent attention and effective management.

Thus, while a majority of previous studies have been based on the consequences and effects of job burnout and experiences of violence among medical personnel, it is clearly more relevant for a health system-building perspective to focus on the causes of burnout and violence in terms of the personality traits of healthcare personnel who are victims of violence. However, to the best of our knowledge, our study is the first to combine emotional intelligence level, experiences of workplace violence, and job burnout levels of healthcare workers fill the research gap of emotional intelligence of medical staff in medical practice. Importantly, China is a developing country with rapid economic and medical development, the number of physicians in China increased by 60% in the past two decades, yet the number of patient visits and the inpatient admissions increased by 276 and 355%, respectively in the mean (26). The surge in health care demand, the weakness of the primary health care system, and the increasing responsibilities demanded by health reform are considered to be the main drivers of the rise in job burnout among health care workers (27). Other countries are or will bear the consequences of healthcare workers' job burnout in the near future, so the findings are internationally representative, serve as a warning, and provide and can be utilized as an important example for other countries.

Hypothesis 1: Emotional intelligence is related to job burnout and workplace violence among healthcare workers.

Hypothesis 2: Workplace violence plays a mediating role in the relationship between emotional intelligence and job burnout levels of healthcare workers.

## Methods

### Design, Samples, and Setting

Six tertiary public hospitals were selected in three provinces (cities) in Eastern (Shandong and Tianjin) and Western (Gansu) China. All study staff received uniform training and passed an assessment before the start of the survey. Permission for this study was obtained from relevant departments, hospital administrators, the medical dispute department, the human resources department, and the respondents. Based on feedback from hospital management experts and mental health professionals, the final questionnaire applied in this paper was validated. The inclusion criteria of this study were as follows: (1) physician, nurses, and medical technicians working in the hospital; (2) more than 1 year of experience; and (3) voluntary participation. The exclusion criteria were as follows: (1) health care workers who were unwilling to participate in the investigation and (2) refresher health care workers and interns. A total of 2,450 questionnaires were distributed, with 2,061 valid questionnaires equaling a valid return rate of 88.95 %, of which 825 healthcare workers had experienced hospital violence, accounting for 40.03% of the sample. The healthcare workers who were victims of violence were used as the subjects of this paper (N=825). The study design has been self-checked through Strengthening the Reporting of Observational Studies in Epidemiology self-check list.

### Emotional Intelligence Evaluation Scale

We used the Emotional Intelligence Scale (EIS) developed by Wong and Law in 2002 (28), which consists of 16 items and is a self-report assessment tool containing the following four dimensions: (1) Self-emotion Appraisal, (refers to a person's ability to understand and clearly express their emotions), (2) Others Emotion Appraisal (refers to a person's ability to recognize and understand the emotions of the people around him or her), (3) Regulation of Emotion (refers to a person's ability to control his/her emotions and quickly recover from psychological frustration), (4) Uses of Emotion (refers to a person's ability to engage in activities that are constructive to personal performance). Each dimension has four items each and seven levels of scoring are used. Several studies using this scale have reported good validity and reliability in this occupational group. The overall emotional intelligence construct produced a Cronbach's alpha of 0.96.

### Workplace Violence Evaluation Scale

The study used the Workplace Violence Scale, which was jointly prepared by the International Labor Organization, the International Council of Nurses, the WHO, and the International Public Service Organization to assess healthcare workers' experiences with workplace violence (21). The scale contains three dimensions: verbal violence, physical violence, and sexual harassment. Verbal violence includes verbal attacks (insults or the use of other words that undermine human dignity—whether face-to-face encounters or telephone conversations, letters, networks, or leaflets, but no physical contact); physical violence includes physical contact or assault with objects (including punching, kicking, slapping, stabbing, pushing, biting, throwing, twisting arms, or pulling hair); and sexual harassment/violence (sexual assault, rape, or attempted rape). Each item was scored on a 4- point scale, reflecting the frequency of respondents' exposure to hospital violence (0=0 times, 1=1 time, 2=2 or 3 times, 3=more than 3 times). The lowest and highest scores were 0 and 27, respectively. The higher the total score, the higher the frequency of violence. The scale has good reliability and validity and has been widely used in China (29–31). The Cronbach's alpha for the entire questionnaire was 0.83.

### Job Burnout Evaluation Scale

To assess job burnout, the most authoritative job burnout scale, the MBI-GS (Maslach Burnout inventorgeneral Survey) (32), was used. The original MBI-GS scale includes 16 items. When considering the internal consistency and validity, Li (33) deleted one item when the scale was translated into Chinese in 2003. The 15-item MBIGS questionnaire included three dimensions: five emotional exhaustion items (EX), four items of depersonalization (DE), and six items of personal accomplishment (PA). These items were scored on a 7-point Likert scales,from 0 (never) to 6 (always), where personal accomplishment reduction was reverse-scored. In this experiment, a Cronbach's alpha scored a 0.94 in emotional exhaustion, a 0.92 in personal accomplish and a 0.94 in depersonalization.

### Data Analysis

EpiData 3.1 was used for dual data entry to ensure data quality. Blank questionnaires, that is, questionnaires with a lot of missing or incorrect information, were removed. The normal distributions of the continuous variables were verified using Shapiro- Wilk test. Descriptive analysis, Univariate linear regression and Pearson correlation analyses were processed using the Statistical Package for the Social Sciences (version 20.0). Following this, the AMOS version 23.0 was used to estimate SEM, and robust maximum likelihood estimation structural equation models were used to estimate the direct and indirect relationships between emotional intelligence, workplace violence, and job burnout. Significant paths were estimated using standardized regression weights. Model fit metrics included χ^2^/df <5, the root mean square error of approximation (RMSEA <0.08), a goodness-of-fit index (GFI>0.90), and a comparative fit index (CFI>0.90).

## Results

### Demographic Characteristics

A common feature of the six tertiary hospitals participating in the survey was that they had more than 500 beds. These hospitals are preventive medical technology centers with comprehensive medical, teaching, and scientific research capabilities. Self-completed questionnaires were used to collect the demographic characteristics of the healthcare workers, including gender, age, education, marital status, profession, department, years of experience, and daily contact with patients. From the perspective of demographic characteristics, the respondents were mainly female (75.0%), and the majority of the workplace included healthcare workers in the ward (75.2%). Further details are provided in Table 1.

**Table 1:** Demographic characteristics of the whole sample (*N* = 825).

**Variables**	**Category**	***N***	**Percentage (%)**
Gender	Men	206	25.0
	Women	619	75.0
Age group (years)	≤ 30	393	47.6
	31–50	379	45.9
	≥51	53	6.8
Education level	Junior college or below	257	31.2
	College	382	46.3
	Master or above	186	22.5
Marital status	Married	562	68.1
	Single	256	31.0
	Other	7	0.8
Profession	Physician	318	38.5
	Nurse	435	52.7
	Medical technician	72	8.7
Department	Emergency	82	9.9
	Surgery	123	14.9
	Ward	620	75.2
Years of experience	≤ 4	306	37.1
	5–10	272	33.0
	≥11	247	29.9
Daily contact with patients	≤ 4	62	7.5
	5–7	380	46.1
	≥8	383	46.4

### Differences Between Participant Characteristics and Scores on Variables

Univariate linear regression results show that there were significant differences in the effects of gender, work sector, and years of experience on the level of exposure to WPV. Among the subjects, emotional intelligence was significantly higher in women than in men. There were significant differences in the effects of gender, education level, marital status, profession, and years of experience on job burnout. Further details are provided in Table 2.

**Table 2:** Univariate analysis and description of each scale.

**Variables**	**Workplace violence**	**Emotional intelligence**	**Job burnout**
Gender			
Male	5.35 ± 4.74	73.56 ± 19.51	37.24 ± 12.94
Female	3.93 ± 3.43	76.65 ± 16.87	34.91 ± 13.85
F/t ^a^	4.64**	−2.05*	2.38*
Age group (years)			
≤ 30	4.14 ± 3.95	76.59 ± 16.24	35.70 ± 13.26
31–50	4.29 ± 3.49	75.38 ± 18.40	35.74 ± 13.81
≥51	5.32 ± 5.21	75.00 ± 21.28	33.51 ± 15.51
F/t ^b^	2.21	0.53	0.64
Education level			
Junior college or below	4.20 ± 3.95	76.18 ± 17.15	34.01 ± 13.31
College	4.38 ± 3.90	75.95 ± 17.84	35.20 ± 14.05
Master or above	4.29 ± 3.85	75.53 ± 17.80	38.46 ± 12.96
F/t ^b^	0.22	0.07	6.03**
Marital status			
Married	4.37 ± 3.77	75.66 ± 18.50	36.13 ± 13.76
Single	4.10 ± 4.03	76.62 ± 15.28	34.13 ± 13.13
Other	4.71 ± 3.73	75.93 ± 17.60	43.29 ± 20.70
F/t ^b^	0.47	1.26	3.03*
Profession			
Physician	4.50 ± 3.97	75.35 ± 18.59	37.03 ± 14.12
Nurse	4.20 ± 3.75	75.99 ± 16.87	34.84 ± 13.09
Medical Technician	3.86 ± 3.85	78.07 ± 17.48	33.49 ± 14.55
F/t ^b^	1.02	0.71	3.27*
Department			
Emergency	5.59 ± 3.90	72.06 ± 19.09	34.45 ± 12.59
Outpatient	4.63 ± 4.43	75.62 ± 20.08	34.80 ± 14.57
Ward	4.04 ± 3.68	76.50 ± 16.82	35.87 ± 13.62
F/t ^b^	6.48**	2.33	0.61
Years of experience			
≤ 4	3.74 ± 3.30	76.82 ± 16.43	34.82 ± 13.26
5–10	4.87 ± 4.28	74.45 ± 17.56	37.74 ± 13.08
≥11	4.33 ± 3.89	76.45 ± 18.96	34.10 ± 14.53
F/t ^b^	6.34**	1.47	5.40**
Daily contact with patients			
≤ 4	3.55 ± 3.04	77.50 ± 15.26	33.10 ± 12.89
5–7	4.44 ± 4.00	74.71 ± 17.78	36.12 ± 13.30
≥8	4.25 ± 3.80	76.88 ± 17.74	35.28 ± 14.11
F/t ^b^	1.46	1.73	1.59

* *p <0.05;*

** *p <0.01.*

a *Statistics were estimated by t-test.*

b *Statistics were estimated by ANOVA*.

### Types of Violence for Victims of Violence

The percentage of violently victimized healthcare workers who had experienced verbal violence in the past 12 months was as high as 98.18%, the highest among all types of WPV in hospitals; physical violence and sexual harassment accounted for 33.82 and 13.21%, respectively.

### Correlations Between Study Variables

As shown in Table 3, emotional intelligence was significantly negatively associated with all three dimensions of job burnout (*p* <0.01) and exposure to workplace violence (*p* <0.01). Workplace violence was significantly and positively associated with two sub-dimensions of job burnout (emotional exhaustion and depersonalization) (*p* <0.01).

**Table 3:** The Pearson correlation analysis among research variables.

**Variables**	**M ±SD**	**1**	**2**	**3**	**4**	**5**
Emotional intelligence	75.93 ± 17.60	1				
Workplace violence	4.29 ± 3.85	−0.22**	1			
Emotional exhaustion	13.04 ± 6.34	−0.18**	0.31**	1		
Depersonalization	8.00 ± 5.09	−0.24**	0.33**	0.69**	1	
Reduced personal achievement	14.53 ± 8.71	−0.24**	0.03	−0.09*	0.11**	1

* *p <0.05;*

** *p <0.01*.

### Mediation Regression Models of Study Variables

Based on Pearson correlation coefficients, two measurement models were developed for emotional intelligence, workplace burnout and emotional exhaustion, and depersonalization (incorporating meaningful demo-graphic characteristics in the single- factor test results: gender, education, marital status, profession, years of experience into the mode), and the models were tested using a likelihood estimation. Since bootstrap methods have the most precise confidence intervals for indirect effects, we used the bootstrap estimation procedure (34) (using a specified bootstrap sample of 1000) to test the significance of the mediating effect of workplace violence on the relationship between emotional intelligence and job burnout.

**Figure 1:**
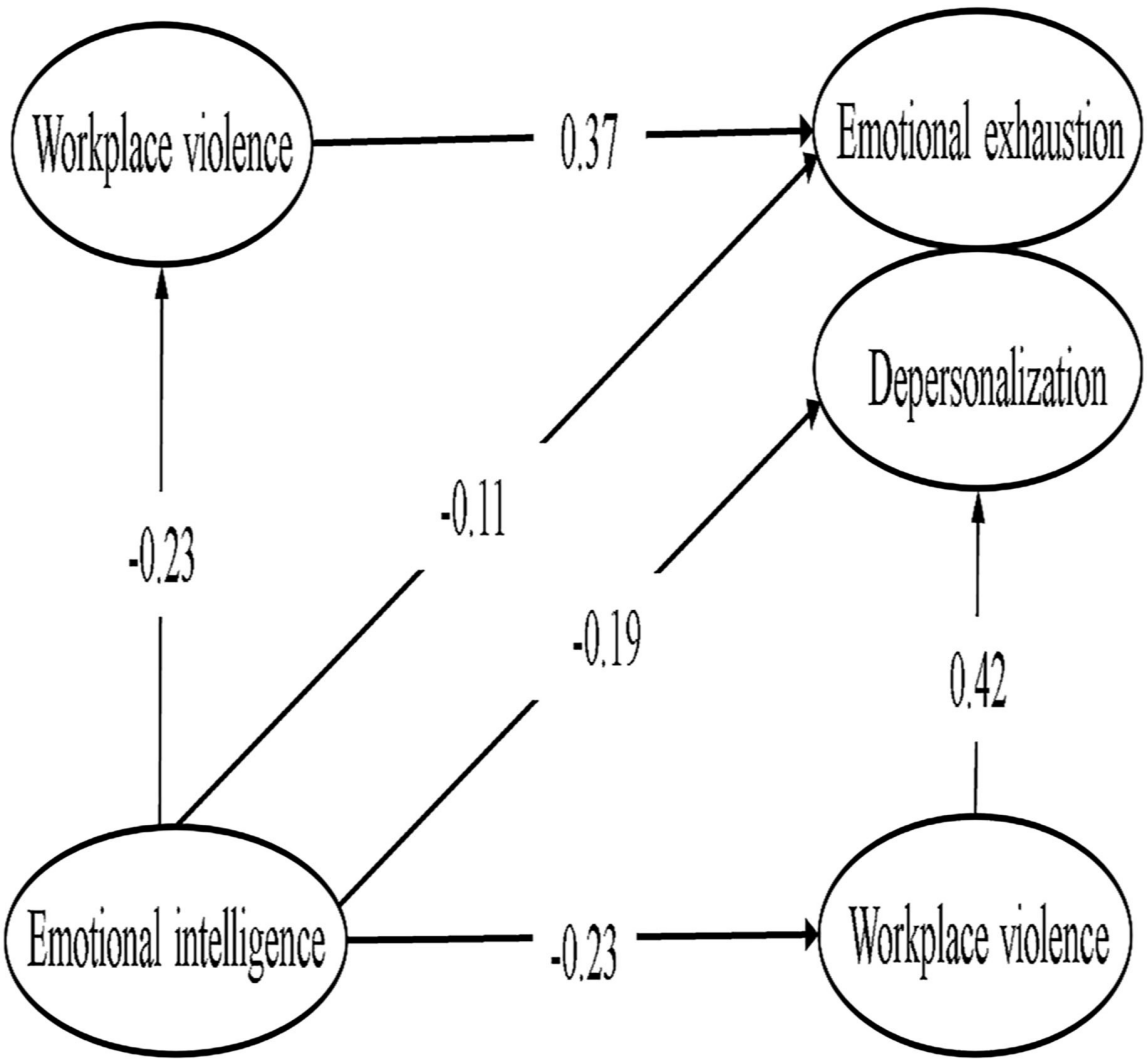
Workplace violence play a mediating role between Emotional intelligence and Emotional exhaustion, Depersonalization.

In the model constructed for emotional intelligence, workplace violence, and emotional exhaustion, the final model fit was good after adding two correlated errors to improve the model fit: CMIN/df = 3.098, RMSEA = 0.050, GFI = 0.969, and CFI = 0.974. The final model plots, the standardized estimate critical ratio, standardizing effects, and mediating effect ratio for the route analysis are detailed in Table 4 and Figure 1, with mediating effects accounting for 45.0% of the total effect.

**Table 4:** Direct and indirect effects and 95% confidence intervals for the final model.

	**95%CI**		
	**Estimated**	**Lower bound**	**Lower bound**
**Emotional intelligence → **			
**Workplace violence → **			
**Emotional exhaustion**			
Total effect	−0.20**	−0.25	−0.10
Direct effect	−0.11**	−0.17	−0.02
Indirect effect	−0.09**	−0.12	−0.05
**Emotional intelligence → **			
**Workplace violence → **			
**Depersonalization**			
Total effect	−0.29**	−0.32	−0.18
Direct effect	−0.19**	−0.23	−0.10
Indirect effect	−0.10**	−0.13	−0.05

* *p <0.05;*

** *p <0.01*.

In the models of emotional intelligence, workplace violence, and depersonalization, after adding two correlated errors to improve the model fit, the model fit was CMIN/df = 2.669, RMSEA = 0.045, GFI = 0.973, CFI = 0.980. The final path coefficients and model plots are detailed in Table 4 and Figure 1, with the mediating effect accounting for 34.48% of the total effect.

In these analyses, if the 95% CI did not include 0, the mediating effect was deemed to be statistically significant.

## Discussion

### Variable Description and Differences

The mean job burnout score of the medical personnel subjected to violence (35.56 ± 13.66) in this study,which was higher than that of the medical personnel not subjected to violence (32.70 ± 12.90). Among the population subjected to workplace violence, 67% of them considered the burnout score to be above 30 and 3% considered the burnout score to be above 60. This shows that moderate burnout is common among healthcare workers subjected to violence, and some participants even experienced high levels of burnout. According to research results, while attaching importance to humanistic care for the physician community, it is especially important to pay more attention to men, highly educated individuals, medical and nursing staff, and healthcare workers with 5–10 years of practice in order to help them reasonably cope with the reality of high expectations and stressful dilemmas through appropriate means such as psychological counseling, group counseling, and concern for specific difficulties in their work lives.

Our results show that women's EI is significantly higher than that of men, which is consistent with Brackett's research results (35). In the analysis of the results of our study it is believed that, compared to men, women's more delicate nature allows them to better perceive and pay attention to the emotions of others and react in a timely manner; thus, they demonstrated a higher level of emotional intelligence. While BarOn's study (36) showed that the difference in emotional intelligence between men and women may be due to the theoretical framework, the selection of measurement instruments at the time of the study, and the difference in the ratio of men to women, gender differences in emotional intelligence need to be further confirmed.

A cross-sectional study of the entire population of medical personnel found that the prevalence of WPV in the past year was 40.03%, which is similar to contemporaneous studies in China. At the same time, other studies have shown that severe WPV in China mainly occurs in tertiary hospitals (67.9%) (37). In our findings, men were more exposed to violence than women; the level of violence victimization was greater in emergency than outpatient clinics, with inpatient units experiencing the least. Regarding work experience, healthcare workers with 5–10 years of work experience had the highest level of violence. Verbal aggression predominated over other forms of violence, similar to other studies conducted in China (38). Our findings support the implementation of a “safe hospital” policy that demonstrates a zero tolerance for workplace violence against healthcare workers, including medical technologists. Specific measures have been described in detail in Vento's study (39).

### Emotional Intelligence Affects Job Burnout

Through empirical research, this study found that emotional intelligence is significantly negatively correlated with the three dimensions of job burnout, verifying Hypothesis 1, which is consistent with previous research results (40, 41). According to Gross's model of emotion regulation, emotions are adaptive behavioral and psychological coping tendencies generated by situations (42). Its basic characteristics specifies that emotions can be regulated, controlled, and managed. Mayer and Salovey (43) proposed that, in theory, emotional intelligence includes the ability to recognize, understand, control, and regulate the emotions of oneself and others. This means that when people with high emotional intelligence encounter negative events, they can first accurately assess and express their emotional state by being acutely aware of their own and the patient's emotional changes, reducing the likelihood of long-term negative emotions building up. On this basis, enhanced emotional management capabilities can better regulate the impact of emotional stimulation at work by strengthening, reducing, prolonging, or ignoring some emotions. This allows the individual to effectively and promptly cope with negative emotions (e.g., anxiety, depression, disappointment) using cognitive and behavioral strategies. Additionally, these individuals tends to spend less energy on emotional regulation and have less emotional stress at work. Therefore, good self-emotional use skills can help healthcare workers know how to recover from frustration, adopt a more positive way to cope with difficult situations, and maintain a stable emotional state to fundamentally maintain the motivation of healthcare workers to work, thus reducing the frequency and extent of job burnout. On the other hand, today's organizations generally draw results from organizational management experiences with high performers that are not necessarily those with outstanding skills or high IQ, but who are good at managing their own relationships with others (44); thus, emotional regulation is considered an important reference indicator of high performers. As a result, individuals with high emotional intelligence tend to be more likely to be promoted as they often build better interpersonal relationships, and thus experience less job burnout.

Soto-Rubiet al. (45) used nurses as research subjects to prove that emotional intelligence has a protective effect against the negative effects of psychological risks. In a 2-year longitudinal study, Gerits et al. found that female nurses with higher EI levels had fewer symptoms of burnout than female nurses with low EI characteristics (46). Karahan et al. confirmed in an experimental study that there is a causal relationship between trait EI and burnout levels; that is, when trait EI increases through training, burnout symptoms decrease (47). Supported by existing theories and research results, we have reason to believe that the emotional intelligence level of healthcare workers (including doctors, nurses, medical technicians) can be used as a protective factor in reducing job burnout.

### The Mediating Role of Workplace Violence in Emotional Intelligence and Job Burnout

The results of this study show that workplace violence has a significant mediating effect on emotional intelligence and the two sub-dimensions of job burnout (emotional exhaustion and depersonalization), partially confirming the original hypothesis 2.

This study reports that the emotional intelligence of medical personnel significantly predicts workplace exposure. Goleman suggested that individuals who can assume a sense of emotional or self-control are able to handle conflicting circumstances more effectively, as a sense of emotional or self-control is very similar to the key components of the emotional intelligence model (48). Mavroveli et al. have shown that individuals with high emotional intelligence solve problems more positively (16). Morrison reports that “there is a negative relationship between the accommodating conflict-handling style and emotional intelligence (49).” Weng et al. found that the higher the emotional intelligence of healthcare workers, the more harmonious their doctor-patient relationship is (50), and a better doctor-patient relationship inevitably leads to a decrease in the number of violent incidents. These findings support our speculation about the plausibility of the relationship between emotional intelligence levels and the frequency of exposure to violence. Başogul et al. (51) used Turkish psychiatric nurses as participants and showed that high emotional intelligence and autonomous personality characteristics reduced the rate of exposure to violence. To our knowledge, this is the first study to examine the relationship between emotional intelligence and workplace violence in the whole population of health care workers (including doctors, nurses, medical technicians).

Healthcare work is a special occupation that requires high emotional and communication skills because medical activity itself requires the full attention of the staff. Excessive concentration can also lead to neglect of workers' emotional output, which affects their attitude toward consultation, listening, patience, communication awareness, and emotional awareness. Studies have demonstrated that nurses' level of emotional intelligence influences the characteristics of the nurse-patient relationship and determines the rate of exposure to violence during conflict (52). In terms of the dimensions included in emotional intelligence, emotionally intelligent healthcare professionals have the ability to identify their own emotions and their patients' feelings and emotions during patient-physician communication, and to appropriately identify and differentiate between types of emotions to assess the potential for violence. By perceiving and understanding the patient's emotions, understanding the patient's suffering, and communicating well with the other party. Examples of this include discussing sensitive and heavy issues with acutely ill patients or their family members, understanding and controlling the emotions of others as much as possible, providing psychological support for the patients, and obtaining patients' cooperation and recognition. Through the perception of the patient's emotional changes, self-protection can be carried out in a timely manner, and methods to ease the patient's emotions are adopted to reduce conflicts with the patient, thereby obtaining the patient's cooperation and acceptance, and reducing the chance of conflict. By effectively regulating and changing their own emotional reactions, the patient's bad emotions can be induced and unblocked in a timely manner so that the patient can have the expected impression of himself. By using this emotional knowledge to guide his or her thinking and behavior, translating the concept of intrinsic humanism into practical action, and being able to manage painful emotions and control impulses in situations involving conflict (20, 52), social capital can be built in an emotionally stable manner, thus reducing the likelihood of violence.

Emotional intelligence at the individual, family, community, and societal levels has been proposed as a cognitive-behavioral strategy in order to overcome violence. Therefore, developing EI capabilities and understanding how to effectively handle conflicts is necessary for anyone, especially healthcare workers who are constantly dealing with stressors in the workplace. Littlejohn showed that the use of emotional intelligence to reduce workplace stress and workplace violence has become the missing link in the nursing and healthcare industry, and emphasized that all healthcare professional leadership must coalesce and insert into all health care practice, education, and operations a focus on EI and WPV—lateral, vertical, or otherwise—and use awareness of EI and its inherent ability as a tool to reduce stress and thus WPV (53).

This study reported that workplace violence has a significant mediating effect on emotional intelligence, emotional exhaustion, and depersonalization dimensions. Similar to the results of this study, other studies have shown that physical and other forms of aggression are associated with higher levels of emotional exhaustion (54) and depersonalization (55). Job burnout is a continuous sequential conceptual model (56). In this process, one dimension promotes the development of the other. We analyze the impact of workplace violence based on the empirical stage model proposed by MBI. first, according to the theory of resource preservation, individuals continue to suppress their inner negative emotions after experiencing workplace violence. This causes individuals to consume a lot of effective psychological resources, and continuous consumption will inevitably lead to exhaustion. Later, when healthcare professionals realize that they need to suppress their negative emotions, they try to keep themselves away from others (depersonalization) by defensive coping and reducing their emotional involvement in order to avoid continued conflict. Depersonalization is mainly an attitude and behavior. As the last part of the job burnout stage model, a reduced sense of achievement involves a negative evaluation of the self, which is caused by the lack of enthusiasm for work due to the long-term influence of the individual who finds that there is a large gap between his or her actual work status and the expected level, making it difficult to complete personal work tasks. After the experience of exposure to violence, there are still many other influences that affect the level of achievement, such as the attributed perceptions of medical personnel and the occurrence of violence over the past year. It is reasonable to assume a non-significant correlation between the level of exposure to violence and a reduction in achievement, both in terms of time and the order of the theoretical model. Furthermore, according to the theoretical model of emotional intelligence, emotional intelligence can reduce job burnout levels by reducing violence. It is also possible to relieve painful emotions in a timely manner through a higher level of emotional intelligence after healthcare staff are subjected to violence and to return to a positive and stable mood in time, reducing the possibility of serious job burnout consequences.

### Possible Measures to Develop the Emotional Intelligence of Healthcare Workers

Hartel et al. feel that the scope for future research in the field of emotions in workplace settings provides the potential to improve substantially our understanding of people and behavior as well as organizations (57). Knight et al. suggested the necessity and feasibility of improving emotional intelligence in public health (58). While their results provided inspiration for our research, they also made members of our research team aware of the importance of focusing on the emotional intelligence of healthcare workers who are more closely connected with patients. In the current context, when healthcare workers and patients are confronted, healthcare workers prefer to adopt a passive conflict-handling model in the process of conflict handling; the use of personal trait development to reduce the occurrence of negative events is undoubtedly beneficial.

Studies (59) that have adopted an ability-based model (28, 60) for EI suggest that EI is a facet of intelligence that is mildly correlated with general mental ability and that it develops mentally in nature. Further, EI increases with age and life experience (61). Research has proven that emotional intelligence is an ability that can be improved through training. Therefore, developing emotional intelligence to help healthcare workers reduce job burnout levels is an urgent issue that every healthcare organization and system needs to address.

For medical institution managers, they should pay attention to the “emotional intelligence” training of the health care worker. A series of measurement tools based on emotional intelligence can be incorporated into the organizational system and can be used as an important indicator in the entire process of career planning for the selection, deployment, training, and promotion of healthcare workers.

Healthcare staff should carry out continuous emotional intelligence training throughout their careers. Utilizing an emotional intelligence improvement course, the specific profile and dimensions of emotional intelligence are developed, such as Reshetnikov et al. proposed “Health Leadership Factory” for public health professional development (62), to help individuals master good expression skills and doctor-patient emotional communication. Additionally, the course helps to reduce the indifference, numbness, and other negative emotions and dehumanization experiences in the interaction with patients under heavy work pressure, This will bring healthcare workers a pleasant emotional state and establish a good self-perception, and it is also helpful in improving the sense of accomplishment at work. Overall, it will help individuals to obtain rich social support in time when they encounter difficulties at work, to improve their adaptability to work and life through the establishment of satisfactory interpersonal relationships, thus increasing their enthusiasm for work and patient satisfaction, and reducing the incidence of work burnout and violence.

Experiential interpersonal interaction training through the use of feedback and manipulative methods such as the Balint Group Method to improve emotional intelligence (63). Healthcare personnel personally experience negative communication events by role swapping and role playing. This allows them to analyze and experience the reasons for the occurrence of various cases and the emotional change states of the patients; thus, discovering the deficiencies in the communication process in terms of cognitive self-emotional regulation. Overall, healthcare workers attempt to better understand, respect, and accept patients' emotions and behaviors; to enhance their own emotional intelligence and communication skills in practice; to reduce the generation of non-technical conflicts; and to help physicians cope with increasingly important professional emotional intelligence needs.

In short, targeted cultivation of the emotional intelligence of healthcare workers is necessary and feasible for the improvement of the doctor-patient relationship, the physical and mental health of healthcare workers, and the construction of health organizations.

## Conclusions

Our study is the first to combine emotional intelligence level, experiences of workplace violence, and job burnout levels of healthcare workers. The structural equation model verified our prediction that emotional intelligence is related to job burnout and workplace violence among healthcare workers, workplace violence mediates between emotional intelligence and job burnout. In light of these results, we believe that the government should pay special attention to preventive measures and strengthen the training of healthcare workers on emotional intelligence; hospital administrators should also pay attention to this issue, and healthcare workers themselves should pay attention to the application and practice of “emotional intelligence”.

## Limitations

(1) This research is a horizontal study that reveals the state of the research object at a certain moment in time. Although the predictive effect of emotional intelligence on the level of burnout and violence is discussed through theories, the presentation of weak correlations between emotional intelligence and other variables (*r* <0.3) (64), more empirical research is needed in order to use positive and reliable methods to provide evidence to further verify the actual mechanism of action. EI may benefit job burnout and violence reduction, as evidenced by follow-up surveys. (2) Additionally, we collected data on whether the healthcare workers experienced WPV in the past 12 months. Therefore, there may be a recall bias in the results, yet the reliability and validity of the scale has been verified by other scholars in this study. (3) Although the Workplace Violence Scale reported good reliability and validity in this study, this scale uses the frequency of violence as a measurement index, and the severity of violence cannot be measured. The development of more reliable research tools will also be our future research field.

## Data Availability Statement

The raw data supporting the conclusions of this article will be made available by the authors, without undue reservation.

## Ethics Statement

The studies involving human participants were reviewed and approved by the Ethics Committee of the School of Public Health of Harbin Medical University (Project Identify Code: HMUIRB20180305). The patients/participants provided their written informed consent to participate in this study.

## Author Contributions

YC and LG responsible for research design, data analysis, and wrote the manuscript. LF provided help with the investigation and data collection. MJ and YL provided guidance in article structure and result interpretation. YM provided assistance in reviewing the manuscript. All authors contributed to the article and approved the submitted version.

## Funding

This study was supported by the National Nature Science Foundation of China (Nos. 71904036, 71874043, and 72174049).

## Conflict of Interest

The authors declare that the research was conducted in the absence of any commercial or financial relationships that could be construed as a potential conflict of interest.

## Publisher's Note

All claims expressed in this article are solely those of the authors and do not necessarily represent those of their affiliated organizations, or those of the publisher, the editors and the reviewers. Any product that may be evaluated in this article, or claim that may be made by its manufacturer, is not guaranteed or endorsed by the publisher.
